# Dissimilarity of Airway and Lung Tissue Microbiota in Smokers Undergoing Surgery for Lung Cancer

**DOI:** 10.3390/microorganisms8060794

**Published:** 2020-05-26

**Authors:** Lena Reinhold, Andreas Möllering, Sönke Wallis, Emanuel Palade, Kathrin Schäfer, Daniel Drömann, Jan Rupp, Simon Graspeuntner, Klaus Dalhoff

**Affiliations:** 1 Medical Clinic III, University Hospital Schleswig-Holstein/Campus Lübeck, Ratzeburger Allee 160, 23562 Lübeck, Germany; andreas_moellering@web.de (A.M.); Soenke.Wallis@uksh.de (S.W.); Daniel.Droemann@uksh.de (D.D.); mk3@uksh.de (K.D.); 2 Department of Surgery, University Hospital Schleswig-Holstein/Campus Lübeck, Ratzeburger Allee 160, 23562 Lübeck, Germany; Emanuel.Palade@uksh.de; 3 Department of Infectious Diseases and Microbiology, University of Lübeck, Ratzeburger Allee 160, 23562 Lübeck, Germany; Kathrin.Schaefer@uksh.de (K.S.); Jan.Rupp@uksh.de (J.R.); Simon.Graspeuntner@uksh.de (S.G.); 4 German Center for Lung Research (DZL), Airway Research Center North (ARCN), Wöhrendamm 80, 22927 Großhansdorf, Germany; 5 German Center for Infection Research (DZIF), Partner Site Hamburg-Lübeck-Borstel-Riems, 23538 Lübeck, Germany

**Keywords:** respiratory microbiome, lung tissue microbiome, respiratory infection, innate immunity

## Abstract

Human airways are continuously colonized by microaspiration of microbiota. Less is known about the presence, origin and composition of microbiota in the lung parenchyma. In a study of 13 patients undergoing surgery for peripheral lung cancer microbiota composition was comparatively evaluated in upper airway, lower airway and lung tissue samples using 16S rDNA analysis. Bacterial density decreased stepwise from upper to lower airways and tissue. On a taxonomic level upper and lower airway microbiota were similar whereas lung tissue showed marked dissimilarities compared to lower airways that may reflect different environmental conditions shaping local microbiota and host immunity.

## 1. Introduction

Compared to other barrier organs, the lungs are scarcely colonized by microbiota. Microaspiration from the upper airways has been shown to be the major pathway to bacterial invasion of the lower respiratory tract. It remains unclear to what extent microbiota of the lower airways of healthy persons represent a resident microbiota or transient populations that are quickly eliminated by exhalation or the local immune system [1]. In contrast, chronic airway diseases are characterized by colonization with a distinct microbiome. Tobacco smoking, the main risk factor for COPD in developed countries, is associated with modifications of oral microbiota, but does not consistently alter the microbiota in the bronchial tree [2,3]. In stable COPD patients, a decreased diversity and/or a higher proportion of Proteobacteria, including potentially pathogenic genera such as *Haemophilus* and *Pseudomonas*, were observed mainly in advanced stages of disease [4,5,6]. Temporal changes in the airway microbiome with shifts in the relative abundance of taxa at the onset and after treatment of acute exacerbations with antibiotics or glucocorticoids are characteristic features of COPD. These changes are not limited to the introduction of new pathogenic strains but involve large parts of the resident microbiome; neutrophilic and eosinophilic types of exacerbation are associated with different patterns of microbiota composition [7].

A significant role for microbiota is also recognized in lung cancer (LC), another smoking-related lung disease [8,9,10]. Evaluation of cancer tissue and/or tumor free tissue in LC patients showed characteristic patterns of microbiota including a shift to Proteobacteria, Actinobacteria [9,11] and/or Firmicutes [8,11]. Differences in microbiota composition may be due to different cancer subtypes; a decreased alpha diversity was found in tumor samples from squamous cell carcinoma [9], and specific communities with links to LC subtypes were observed with *Acinetobacter*, *Brevundimonas* and *Propionibacteria* in adenocarcinoma and Enterobacteriaceae in squamous cell carcinoma [12]. Greathouse et al. suggested a role of microbiota in carcinogenesis, describing an interaction between smoking, smoking-related TP53 mutations in lung cells and microbiota that are enriched in this environment [13].

Most studies on the “lung” microbiome evaluated samples from the airways retrieved by sputum collection or bronchoalveolar lavage (BAL). Comparative studies confirmed the continuity of airway microbiota along the respiratory tract combined with decreasing density [1,14]. However, it remains unclear if the composition of airway microbiota is representative also for the distal lung tissue, which is of interest since major pulmonary conditions including COPD, LC, pneumonia and ARDS (Acute respiratory distress syndrome) involve the lung parenchyma as well as the airway compartment. The alveoli form a distinct environment characterized by highly specialized epithelia, immune cells, a surfactant rich lining fluid and the absence of mucus, which all affect bacterial growth conditions [14]. Few studies evaluated the pulmonary microbiome using human lung tissue (HLT) [4,8,9,15,16,17,18]. Since no study has directly compared microbiota from BAL samples and from HLT, we evaluated respiratory microbiota composition in patients undergoing surgery for pulmonary nodules using samples from different airway locations and HLT. Thus, we compared respiratory microbiota composition using samples from different airway locations and HLT. The main questions were the bacterial abundance at different levels, similarity of microbiota composition between the compartments and association of microbiota with inflammatory patterns in HLT.

## 2. Materials and Methods

### 2.1. Patient Data and Ethical Declaration

Thirteen patients who underwent bronchoscopic evaluation for pulmonary nodules and received an anatomic lung resection were included in the study. All participants were current smokers or ex-smokers; four of them had a diagnosis of COPD according to Global Initiative for Chronic Obstructive Lung Disease (GOLD) criteria (Table 1). All patients underwent thoracic computer tomography for diagnostic purposes. There were no cases with antineoplastic treatment before surgery. See Table 1 for cancer histology and stage. In 11/13 cases the diagnosis of lung cancer was confirmed by histology; in the remaining two a diagnosis of benign tumor and aspergillosis was made, respectively. Patients were free of clinical signs of infection at the time of sampling. Therapy with inhaled (*n* = 2) or oral (*n* = 1) steroids and/or antibiotics (*n* = 3) had been administered within the last 90 days prior to sampling in some cases.

This study was approved on 4 May 2014 by the ethics committee of the University of Lübeck, Germany (AZ 14-061). All participants granted written informed consent.

### 2.2. Sample Collection

Sterile swabs (Hain Lifescience, Nehren, Germany) were used to sample microbiota from the posterior oropharynx. BAL samples were taken during diagnostic bronchoscopy using a two-scope technique. The first bronchoscope was used for local anesthesia (Xylocain^®^ 2%, AstraZenecaGmbH, London, England) of the pharynx and the glottis. Before inserting the second bronchoscope, it was washed with sterile saline as a negative control. Thereafter, the scope was introduced transorally and wedged in a segmental or subsegmental bronchus before further diagnostic manipulations. The selected segment had to be unaffected by the tumor. Normal saline was instilled and the recovered BAL fluid was immediately centrifuged. The pellet as well as the swabs were stored at −80 °C.

Lung tissue was transported immediately after surgical resection to the department of pathology, where a tumor-free part of the tissue was cut, stored and frozen at −80 °C. All instruments during this process were sterile.

### 2.3. DNA-Isolation

BAL samples were centrifuged in 50 mL Falcon tubes at 16,000× *g* and the pellet was used for DNA isolation. Swabs and human lung tissue (~0.25 g) were directly introduced to DNA isolation. DNA was isolated from sample material using the MoBio PowerSoil^®^ Kit (MO BIO Laboratories, Carlsbad, CA, USA) following the instructions from the manufacturer’s protocol. We introduced 2 h incubation with OB-Protease at 50 °C followed by homogenization of the sample using a MoBio PowerLyzer^®^ (MO BIO Laboratories, Carlsbad, CA, USA), both prior to the first centrifugation step. Isolated DNA was stored at −20 °C.

### 2.4. PCR and Sequencing

We amplified partial 16S gene sequences from isolated DNA using the primer pair V3F/V4R (V3F: 5′-CCTACGGGAGGCAGCAG-3′/V4R: 5′-GGACTACHVGGGTWTCTAAT-3′) to amplify the V3/V4 hypervariable region. Sequencing was performed on a MiSeq sequencer (Illumina, San Diego, CA, USA). All primers contained unique identifier sequences (barcodes) to distinguish between the samples following the approach by Kozich et al. [19]. PCR was performed as follows: 98 °C for 5 min followed by 30 cycles with 98 °C for 9 s, 55 °C for 60 s and 72 °C for 90 s followed by a final step at 72 °C for 10 min. After, PCR samples were stored at −20 °C until further usage. Amplicons were quantified on an agarose gel with a DNA ladder as reference, where the concentration of each amplicon was determined by comparison to a ladder band of the same size and intensity as the respective amplicon. Equimolar amounts of the correct sized fragments were pooled for sequencing. Afterwards, the pool was run again on an agarose gel and eluted with a MinEluteGel Extraction Kit (Qiagen, Venlo, The Netherlands). The pool was stored at −20 °C until sequencing. Sequencing was performed on a MiSeq sequencer (Illumina, San Diego, California, USA) using the MiSeq Reagent Kit v3 (600 cycles), as described by Kozich et al. [19].

### 2.5. Data Processing

Fastq files were processed using Mothur, version 1.38.1 [20]. Contigs were produced of forward and reverse sequences and any sequence was removed if it had ambiguous bases, a homopolymer length > 12 or a size longer than the amplified fragment. We aligned the remaining sequences using a customized SILVA reference data base [21] and removed unaligned sequences. Chimeras were detected using the UCHIME algorithm [22] as implemented in Mothur [20] and removed from the data set. We classified the sequences using the Mothur-formatted Greengenes [23,24] training set, version gg_13_8_99, with a cutoff of 80 and removed non-bacterial sequences. Further analysis was performed using operational taxonomic units (OTUs) clustered with a similarity threshold of 97% or based on taxonomic assignment.

### 2.6. Data Normalization and Decontamination

We used geometric mean of pairwise ratios as the normalization method for zero-inflated sequencing data, a method developed recently to correct for bias introduced by variable sequencing depth [25]. We further ran the decontam algorithm using the frequency-method as implemented in R to assess contamination and decontaminate the sequencing data prior to further analysis [26]. See Table S3 for the removed taxa which were identified as contamination following the decontam algorithm.

### 2.7. qPCR

Absolute bacterial biomass was quantified using primers targeting the bacterial 16S rRNA gene on the basis of a standard curve generated from *E. coli* bacterial counts for samples and isolation controls. PCR was performed as follows: 98 °C (10 min); 45 cycles of: 98 °C (9 s), 55 °C (30 s) and 72 °C (30 s) using the LightCycler 480 SYBR Green Master I Kit (Roche, Rotkreuz, Switzerland) on the LightCycler 480 II device with the corresponding software (release 1.5.0). PCR-reactions were carried out with a volume of 20 µL with 2 µL of template. With the end of the PCR, a melting curve analysis was performed. We used the following primer sequences: 16S forward 5′-AGA GTT TGA TCC TGG CTC AG-3′, 16S reverse 5′-TGC TGC CTC CCG TAG GAG T-3′. Further information on primers and used methodology according to the MIQE checklist [27] is provided in Supplementary Materials Table S5.

### 2.8. Quantitative Cytokine Analysis

To quantify cytokine concentrations in cell free supernatants of lung tissue in vitro, ELISA (IL-18; MBL, Nagoya, Japan) and multiplex-assay (IL1b, IL6, IL8, IL10, IL12, IL17A, G-CSF, GM-CSF, TNF-α; Bio-Rad, München, Germany) were used after incubation of 0.3–0.4 g pieces of lung tissue at 37 °C and 5% CO_2_ in 2 mL RPMI medium (RPMI 1640 + 10% FCS) for 24 h. Cytokine assays were done according to kit instructions and were performed on 11 of the 13 lung specimens due to tissue shortage after pathological assessment in the remaining cases.

### 2.9. Statistical Analysis

Statistical Analysis was performed using SPSS Statistics (Version 23.0, IBM, Armonk, NY, USA) and R Studio (Version 1.0.153, RStudio Inc., Boston, MA, USA). Results are shown as mean ± SEM. Wilcoxon rank sum test with Holm correction for multiple testing was used for testing differences in fold change of bacterial amount in the airways and lung tissue. A Kruskal–Wallis ranksum test was used for testing significant differences in relative read count along the respiratory tract. In the case of significant differences a Wilcoxon ranksum test with Benjamini-Hochberg correction for the number of performed tests was performed for pairwise comparisons. For measuring alpha diversity, we assessed Shannon’s diversity index for each sample using R package vegan. Bray–Curtis indices were assessed and visualized with a principal coordinates analysis. Biplots and heatmaps were assessed using R package ggplot2 [28] and vegan [29]. Cluster analysis based on Euclidean distances (average linkage method) was performed on the proinflammatory cytokines. Differences in cytokine concentration between the two groups were assessed using a t-test with Bonferroni correction for the number of performed tests. SourceTracker [30] was used to estimate the contribution of taxa from proximally located airway compartments and of taxa from unknown origin to the lower airway and lung tissue microbiota.

## 3. Results

**Figure 1:**
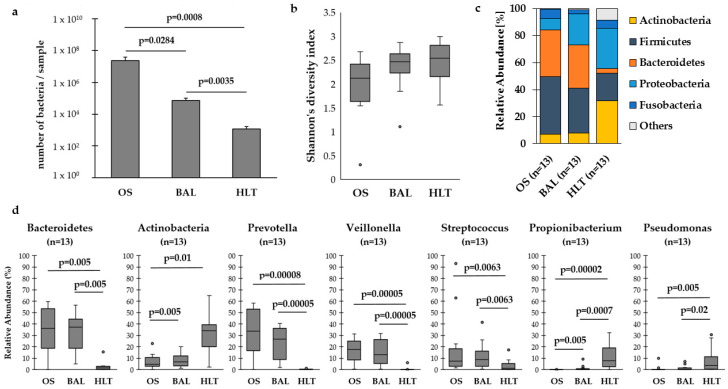
(**a**) Number of bacteria per sample among the respiratory tract. We found significantly lower amount of bacterial cells in samples from the lower airways (bronchoalveolar lavage, BAL) and human lung tissue (HLT) compared to the upper airways (oral swabs, OS). There was also a significant difference between BAL and HLT. (**b**) Shannon’s diversity index was numerically lower in the upper airways. (**c**) The distribution of the five most abundant phyla in the airways and the lung tissue. The mean relative abundance of Bacteroidetes in the airways was significantly larger than in the lung tissue, while Actinobacteria showed a significantly higher relative abundance in the lung tissue. (**d**) Looking at the distribution of the most abundant genera, *Prevotella*, *Veillonella* and *Streptococcus* occurred with a significantly higher mean relative abundance in the upper and lower airways than in the lung tissue. In contrast, *Pseudomonas* and *Propionibacteria* were significantly more abundant in HLT (Outlier are marked with a black dot).

We compared absolute quantification of bacterial mass between the different sample types using qPCR of bacterial 16S rDNA. Figure 1a shows a stepwise decrease of bacterial density from the upper airways to the lower airways and lung tissue. On a taxonomic level, upper and lower airway microbiota showed similar composition dominated by oral taxa such as *Prevotella*, *Veillonella* and *Streptococcus*. In contrast, prominent taxa in HLT were Proteobacteria and Actinobacteria (Figure 1c–d). Supplementary Materials Figure S1 shows rarefaction curves for sample types and Figure S2 the distribution of the five most abundant phyla along the respiratory tract of the 13 patients.

**Figure 2:**
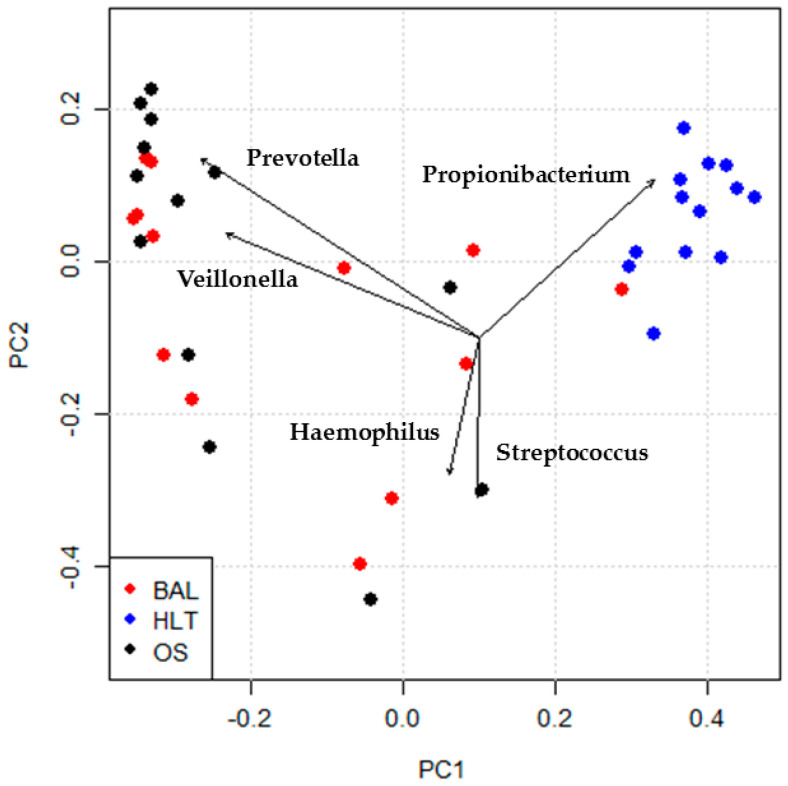
Biplot of principal coordinates analysis on the basis of Bray–Curtis dissimilarities. The lung tissue samples (HLT) and airway samples (BAL, OS) showed a significant difference (Adonis test, number of permutations = 999, *p*-value = 0.001). Arrow length is proportional to the strength of the correlation between taxa and ordination.

Dissimilarities between BAL and HLT microbiota were confirmed by principal coordinates analysis (Figure 2), illustrating that *Pseudomonas* and *Propionibacteria* were associated with HLT, while *Veillonella* and *Prevotella* were associated with BAL. See Supplementary Materials Figure S3 for a heatmap of microbiota distribution.

**Figure 3:**
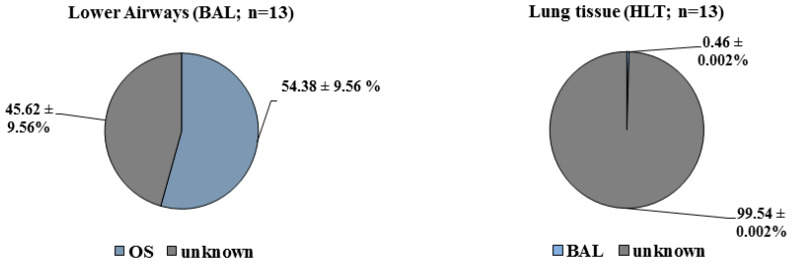
SourceTracker was used to estimate the contribution of taxa from proximally located airway compartments and of taxa from unknown origin to the lower airway and lung tissue microbiota. It shows upper airway as the major source of the composition of the lower airway microbiome. The lower airway contributes just a small proportion to the lung tissue microbiota. The largest part is unknown.

To assess the contribution of proximally located compartments to taxa composition, we used SourceTracker analysis [30] in R. SourceTracker uses an abundance table to estimate the proportion of a given type of samples (“source”) contributing to another sample type (“sink”). We used oral swabs as sources for BAL and BAL as sources for lung tissue. The majority (54.4%) of BAL taxa was derived from upper airways, whereas only 0.46% of HLT microbiota reflected microbiota from BAL; 99.54% of the microbiota in human lung tissue could not be explained by microbiota composition of the upper or lower airways (Figure 3).

Interactions between microbiota and the local host response were assessed by natural clustering of cytokine concentrations in HLT which identified two subgroups differing in a history of antibacterial therapy. Average linkage method based on Euclidean distance was used to calculate distance between elements. Silhouette coefficient was used for selection of the optimal number of clusters. See Supplementary Materials Figure S5 and Table S2 for a cluster dendrogram and average cluster coefficient for the number of clusters. A lower Shannon index and high cytokine concentrations were found in the pretreated subgroup (Supplementary Materials Figure S6). Interestingly, *Haemophilus influenzae*, as a major respiratory pathogen, was mainly identified in BAL and related to release of inflammatory cytokines from HLT (Supplementary Materials Figure S4, Table S1).

## 4. Discussion

This study shows a very sparse and markedly different microbiome in human lung tissue as compared to the lower airways from the same patients. Upper and lower airway microbiota demonstrated a high degree of similarity, and the majority of BAL taxa were derived from oropharyngeal sources, as shown previously [1,14]. In contrast, few taxa from HLT were derived from lower airway microbiota. BAL captures small airways in addition to alveoli, which may explain the difference to HLT samples [17]. Proteobacteria were abundant in the HLT, as observed previously [4,15]. A particular finding of our study was the frequent detection of *Propionibacteria* in HLT, but not in airway samples. While this finding is not consistent through all prior microbiome studies, *Propionibacteria* have been detected repeatedly in lung tissue [16,18]. In sarcoidosis, *P. acnes* has been isolated as the most common commensal bacteria in lymph nodes and peripheral lung tissue [31]. An alternative explanation may be enrichment in *Propionibacteria* in lung cancer tissue, which has been observed recently in patients with adenocarcinoma [12].

Our data are not comparable to studies using explants from patients with endstage lung diseases [4,15,17] or swabs from resected tissue [16], which probably reflects differences in patient populations and methodology.

We are not aware of other studies directly comparing microbiota from BAL with human lung tissue. However, Pragman et al. compared microbiota in swabs from surgically resected lung tissue and central bronchi with upper airway samples of COPD patients. Similar taxa composition between oral swabs and lung tissue was observed; however according to SourceTracker analysis, the majority of HLT taxa were of unknown origin, as in our study [16].

Two recent studies evaluated similar populations as this study using tissue from lung cancer patients undergoing surgery. Liu et al. observed in lung tissue from cancer patients that Firmicutes were enriched whereas Proteobacteria were reduced compared to emphysema patients [8]. In another study using tissue samples from a cancer biobank, *Acinetobacter* was frequently detected [9], which could also be confirmed by our data (Supplementary Materials Figure S3) and others [12]. *Acinetobacter* has been identified in cigarette smoke and might have a role as external source of microbiota in smokers [9].

Limitations of this study include the small sample size, cross-sectional design and heterogeneity of the population, including smokers with and without COPD, although no differences between these subgroups were detected (data not shown), as well as comparing different sampling materials (swab, BAL and tissue). The role of immune cells in shaping local microbiota could not be assessed due to limited availability of tissue.

Laboratory contamination with bacterial DNA is always an issue in experiments with low microbiota concentrations; to exclude bacterial contamination as a source of our data as best as possible, we followed recommended publication standards [32]. We ran isolation controls in parallel for each round of DNA-isolation. These controls comprised all materials used during sample processing except the sample itself. Thus, all devices used for this study were rinsed with buffer to serve as control. The isolation controls were run through PCR, as were all samples. We included only samples into this study if an amplicon of the expected size was visually present, and in addition the respective isolation controls remained visually negative in an agarose gel following PCR. Still, we included the isolation controls into the pooled library at volumes comparable to those used for the actual samples. Following sequencing, isolation controls were processed as described for all samples and used for decontamination of the data set, as described within the Methods section (see also Supplementary Materials Tables S3 and S4). In addition, qPCR results showed that bacterial counts of isolation controls were approximately two log^10^ levels lower than those of the HLT samples (data not shown). Thus, we do not consider contamination an explanation for our results.

In conclusion, our findings suggest the presence of a distinct, very sparse microbiome in lung tissue which may have evolved independently of airway microbiota. This may be due to different growth conditions in the alveolar spaces and is a caveat to relying solely on BAL for representative sampling of alveolar microbiota. Larger studies are warranted to extend these findings and to understand their functional implications on host immunity and the pathogenesis of lung diseases.

## Figures and Tables

**Table 1:** Clinical data of the patient population.

	Age	Gender	Smoking Status/Packyears	FEV1%	FEV1/FVC%	COPD (Stage)	Lung Cancer	Cancer Stage (UICC)
1	74	female	CS ^1^/25	77	74	no	SCC ^3^	IA
2	62	female	CS ^1^/22	51	66	COPD (GOLD II)	AC ^4^	IIA
3	80	male	CS ^1^/60	50	68	COPD (GOLD III)	SCC ^3^	IB
4	68	female	CS ^1^/40	74	106	no	AC ^4^	IIA
5	63	male	ES ^2^/40	72	82	no	SCC ^3^	IIIB
6	58	male	CS ^1^/40	35	50	COPD (GOLD III)	AC ^4^	IIB
7	73	male	ES ^2^/60	87	102	no	SCC ^3^	IIA
8	61	male	CS ^1^/80	75	102	no	SCC ^3^	IIA
9	63	female	ES ^2^/25	88	96	no	LCNEC ^5^	IIA
10	72	male	CS ^1^/n.d.	90	108	no	AC ^4^	IA
11	61	male	ES ^2^/110	42	68	COPD (GOLD III)	other ^6^	no
12	61	male	CS ^1^/30	79	101	no	SCC ^3^	IA
13	50	male	CS ^1^/43	99	87	no	other ^7^	no

^1^ current smoker, ^2^ ex-smoker, ^3^ squamous cell carcinoma, ^4^ adenocarcinoma, ^5^ large cell neuroendocrine carcinoma, ^6^ pleural solitary fibrous tumor, ^7^ aspergillosis.

## Supplementary Materials

The following are available online at https://www.mdpi.com/2076-2607/8/6/794/s1, Figure S1: Rarefaction curve of the three groups, Figure S2: Distribution of the five most abundant phyla along the respiratory tract of the 13 patients, Figure S3: Heatmap of microbiota distribution in the lower airways and lung tissue, Figure S4: Distribution of Haemophilus along the respiratory tract, Figure S5: Cluster dendrogram, Figure S6: Inflammatory cytokines and Shannon’s diversity index according to history of antibiotic therapy, Table S1: Correlation between proinflammatory TNF-α and *Haemophilus influenzae* and *Pseudomonas*, Table S2: Silhouette coefficient for the number of clusters, Table S3: Removed taxa which were identified as contamination following the decontam algorithm, Table S4: 20 most abundant taxa detected in isolation controls, Table S5: Additional information on qPCR-Primers and qPCR conditions. The sequencing data used for this study is available at the European Nucleotide Archive under the accession number PRJEB38158 including metadata.
