# Supplementary material for: Dissimilarity of Airway and Lung Tissue Microbiota in Smokers Undergoing Surgery for Lung Cancer
Source: Microorganisms. 2020 May 26;8(6):794. doi: 10.3390/microorganisms8060794 (PMC7355471; doi:10.3390/microorganisms8060794)
Supplement: Supplementary file 1 [file microorganisms-08-00794-s001.pdf]

Supplement

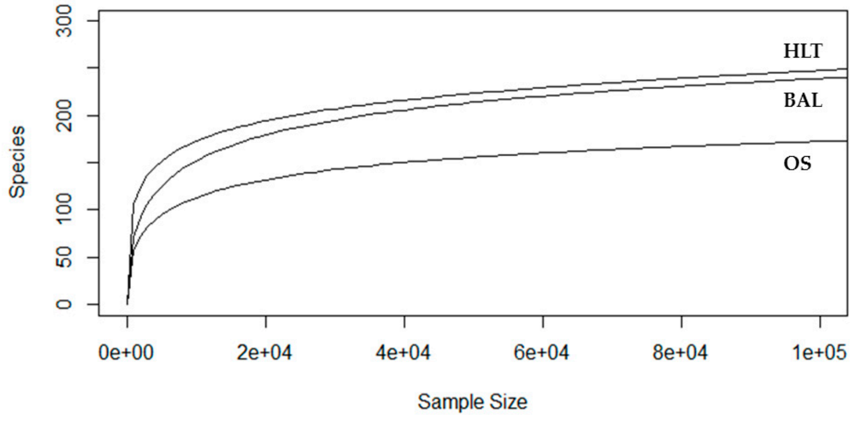

Figure S1: Rarefaction curve of the three groups (OS, BAL, HLT)

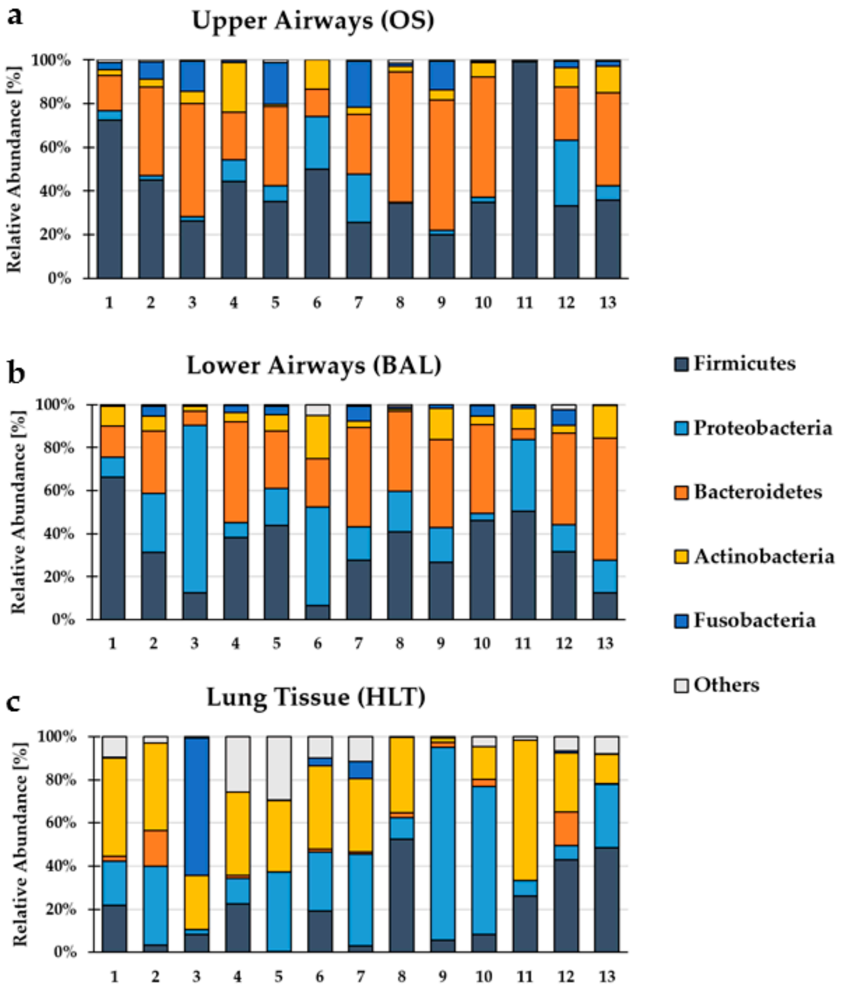

Figure S2. Distribution of the five most abundant phyla along the respiratory tract in the upper airways (a), lower airways (b) and lung tissue (c) of the 13 patients.

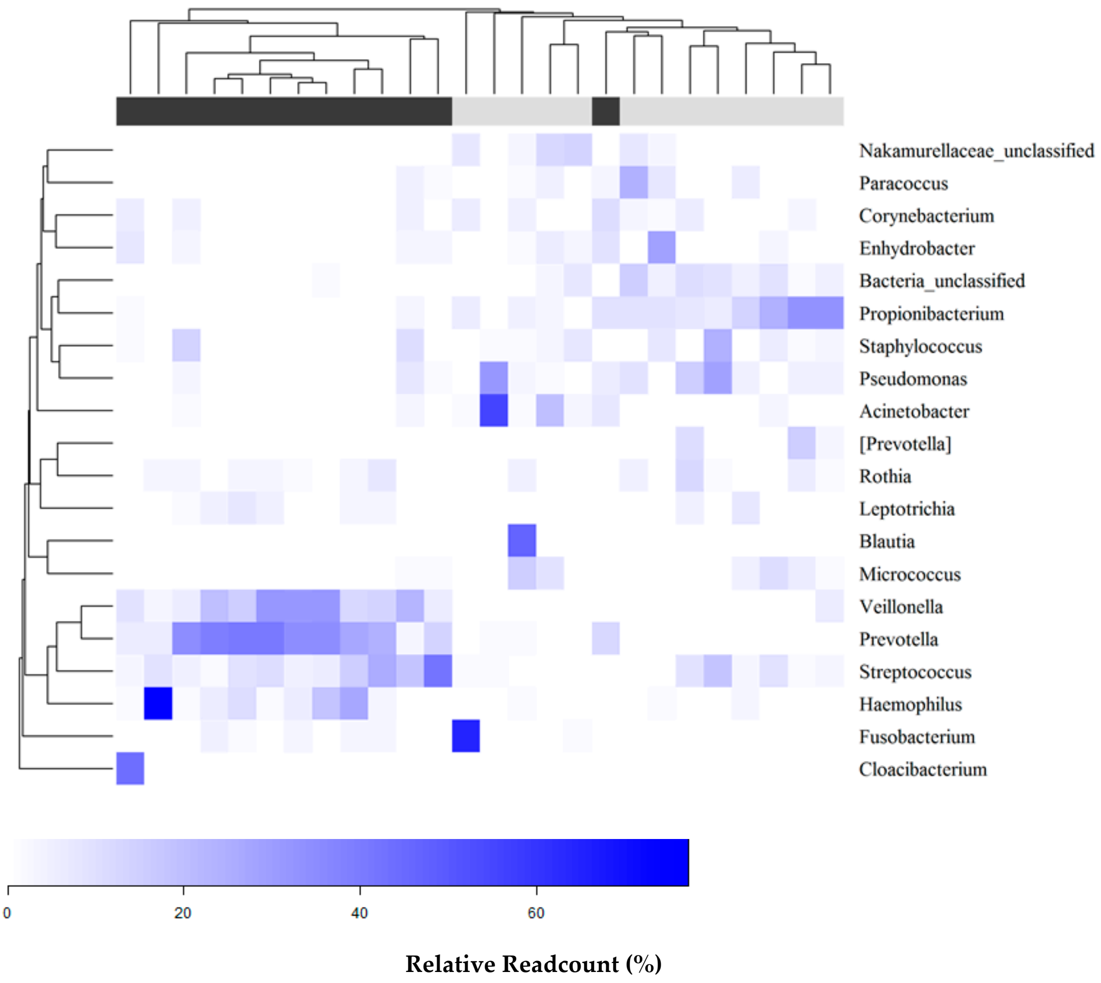

Figure S3. The distribution of genera clearly differs between lower airways and lung tissue. Each column corresponds to the samples of the lower airways (dark grey) and the lung tissue (light grey) from the 13 patients and were clustered hierarchical in two groups. Each row represents a bacterial genus. The 20 most abundant genera in the two compartments are shown (A). The colour key represents the relative abundance of each genus in each sample (B).

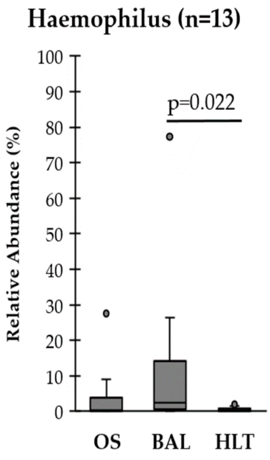

Figure S4: Distribution of Haemophilus along the respiratory tract.

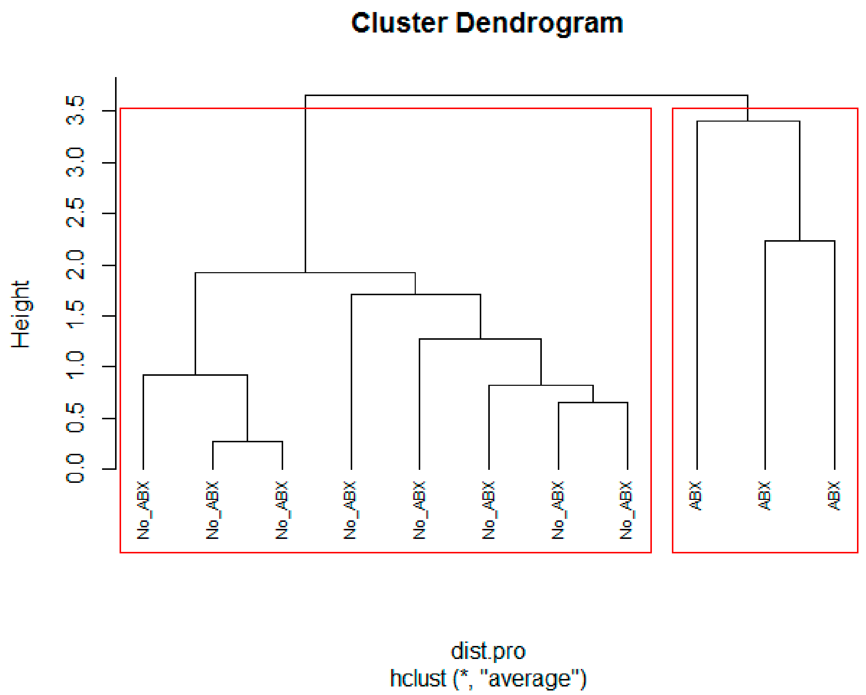

Figure S5: Cluster Dendrogram. (ABX = antibiotic therapy)

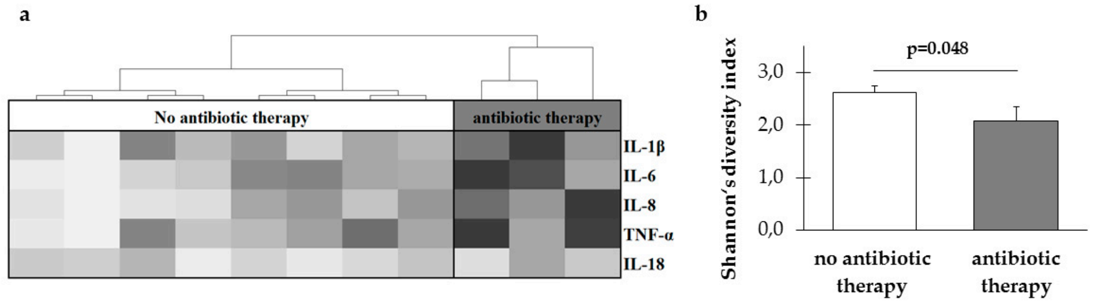

Figure S6: Hierarchical cluster analysis based on Euclidean distance (average linkage method) divides the inflammatory cytokines in two groups. One group had no history of prior antibiotic therapy, while the other group had received antibacterials. Cytokine concentration is higher in the group with antibiotic pretreatment during the last 90 days (**a**), while Shannon's diversity index is significantly lower in this group (**b**). The shade of colour represents the cytokine concentration (the darker the colour shade the higher is the cytokine concentration).

Two genera encompassing important respiratory pathogens were separately considered and showed a divergent distribution pattern: *Haemophilus* are centered in the lower airways, whereas *Pseudomonas* are more frequently detected in the lung parenchyma. We see also a significant correlation between these two taxa and the proinflammatory cytokine TNF-a concentration in the lung tissue (Pseudomonas: R =-0,827, p=0,002; Haemophilus influenzae: R=0,610, p=0,046) (Table S1).

Table S1: Correlation between proinflammatory TNF-α and Haemophilus influenza and Pseudomonas

TNF-α	Spearman coefficient	Significance p	n
Haemophilus influenzae	0,610	0,046	11

Pseudomonas	-0,827	0,002	11

Table S2: Silhouette coefficient for the number of clusters

k (number of cluster)	Silhouette coefficient
2	0.46
3	0.33
4	0.35

Table S3: Removed taxa which were identified as contamination following the decontam algorithm

Removed contaminants
Hymenobacter
Chryseobacterium
Comamonas
Solirubrobacterales_unclassified
Cytophagales_unclassified
Pseudonocardia

Table S4: The 20 most abundant Taxa present in isolation controls after removal of taxa given in table S3 (only samples with > 100 reads were included)

Taxa detected in negative controls
Veillonella
Pseudomonas
Corynebacterium
Staphylococcus
Streptococcus
Enterobacter
Rothia
Enhydrobacter
Anaerococcus
Paucibacter
Enterobacteriae_unclassified
Granulicatella
Kocuria
Actinomyces
Paracoccus
Neisseria
Garciella
Lachnoanaerobaculum
Capnocytophaga
Micrococcus

Table S5: Additional information on qPCR-Primers and qPCR condition:

Primer validation	Efficiency: 96.45% / r^2: 0.99 / slope: -3.038 / y-intercept: 31.95
Limit of detection	6 bacterial counts (*E. coli*)
Amplicon length	348 bp
Method of Cq determination	Abs Quant/Fit Points
Cq of non-template controls	>35
